# Network integration of neocortical Cajal-Retzius neurons shapes early cortical dynamics and contributes to their transient nature

**DOI:** 10.1186/s12964-026-03210-6

**Published:** 2026-09-09

**Authors:** Federico De Rosa, Werner Kilb, Heiko J. Luhmann, Anne Sinning

**Affiliations:** Institute of Physiology, University Medical Center Mainz, Johannes Gutenberg University, Duesbergweg 6, Mainz, 55128 Germany

**Keywords:** Cajal-Retzius neurons, Neocortex, Development, Cell death, Mouse, GABA, Activity, NKCC1

## Abstract

Cajal-Retzius neurons (CRN) represent an early-born transient neuronal population in the mammalian neocortex. They are best known for secreting reelin, which is essential for neuronal migration and layer formation in the developing cortex. However, their functional integration into developing cortical circuits and their contribution to early network dynamics have been poorly understood. In this study, we investigated the structural and functional integration of CRN into developing neocortical networks with immunohistochemistry, three-dimensional reconstruction of GABAergic inputs, extracellular electrophysiology and calcium imaging. In addition, multiplexed FISH was used to determine the expression levels of the chloride transporters NKCC1 and KCC2 in CRN at different postnatal stages. Finally, we employed optogenetic stimulation to assess the functional and developmental consequences of CRN activation. We found that CRN receive dense dendritic GABAergic synaptic inputs at early postnatal stages. Functional analyses revealed that a substantial fraction of CRN is spontaneously active, and that their activity is synchronized with both CRN and non-CRN neurons. At the molecular level, they display a persistent NKCC1-dominant expression profile. This profile can contribute to the excitatory GABA responses, but also affect their cell death, as chronic blockade of NKCC1 was associated with reduced CRN loss in vitro. Acute optogenetic activation of CRN at early stages increased network excitability in organotypic neocortical cultures and induced stimulus-evoked field responses, while chronic activation at later time points accelerated CRN loss. Overall, these findings demonstrate that, during development, dense GABAergic input connectivity, coupled with the putative excitatory action of GABA, can engage CRN in early network activity. Furthermore, not only do CRN actively participate in early immature circuits, but their activity also amplifies spontaneous network dynamics and contributes to their own transience, highlighting a potential novel functional role of CRN during neocortical development.

**Supplementary Information:**

The online version contains supplementary material available at https://doi.org/10.1186/s12964-026-03210-6.

## Introduction

Cajal-Retzius neurons (CRN) represent a unique cell type and despite having been described by Santiago Ramón y Cajal and Gustaf Retzius more than a century ago, a comprehensive understanding of this neuronal population is still lacking [1, 2]. CRN are amongst the earliest generated neurons of mammalian cortex and, together with subplate neurons, they represent one of the main transient neuronal populations in the neocortex [3]. In mice, CRN are born between embryonic days (E) 10.5 and 12.5 [4, 5], while in humans they have been observed from the fifth gestational week [6]. Neocortical CRN originate from different regions of the pallial border reflecting their heterogeneity into distinct subtypes [7]. From their origin site, CRN migrate tangentially across the telencephalon surface and settle in the marginal zone, the future layer I (LI) of the neocortex [8]. CRN almost entirely disappear from murine cortex in the third postnatal week, with the end of radial migration [9–11]. In humans, despite a massive reduction between gestational week 23 and 28, a substantial number of CRN persists until adulthood [12]. CRN subpopulations not only differ in their origin, but also in their migration routes, functions and molecular identity [2]. The transcription factor p73 (encoded by *Trp73*), and particularly its ΔNp73 isoform, is expressed in most cortical CRN subpopulations [13, 14]. This heterogeneity also extent to the mechanisms controlling CRN death. Notably, survival of septum-derived ΔNp73-positive CRN was shown to be influenced by electrical activity, as well as by genetic inactivation of the pro-apoptotic factor *Bax* [15, 16]. Typical CRN are characterized by a peculiar morphology (ovoid soma, bipolar configuration with a thin axon opposed to a thick dendrite) and typical electrophysiological features (low resting membrane potential, high input resistance, small amplitude and long duration of action potentials) [1]. One of the best described functions of CRN during neocortical development is their release of the extracellular matrix protein reelin, that plays a critical role as a positional signal in radial migration and structural lamination of the cortical layers [17, 18]. Previous retrograde tracing experiments identified as CRN presynaptic partners layer V pyramidal neurons and inhibitory interneurons in layers I and VI [19, 20]. While the extend and functionality of glutamatergic synapses on CRN are still under discussion [21–24], GABAergic input connectivity has been better characterized [25, 26]. Supporting the existence of functional synapses on CRN, electrical stimulation in LI elicited somatic calcium transients in CRN [27] and GABA_A_ receptor-mediated spontaneous postsynaptic currents were recorded from CRN [21, 28, 29]. With respect to their output connectivity, neocortical CRN have been shown to innervate LII/III and LV pyramidal neurons as well as other CRN within LI. However, the synaptic function of these connections remains elusive [23, 30]. Thus, in contrast to the well-studied connectivity of most neuronal populations, such as immature pyramidal neurons [31, 32], the input-output organization of CRN and their roles within immature cortical networks during postnatal development remain poorly characterized. The aim of this study therefore was to investigate the structural and functional integration of CRN into developing neocortical networks, the expression profile of the chloride transporters that may underlie their excitatory response to GABA, and how it changes with development, and the functional implications of CRN activation in immature cortical networks. First, we conducted a longitudinal analysis of dendritic GABAergic synapses onto cortical ΔNp73-positive CRN to examine the input connectivity of CRN and compared them with the general maturation of GABAergic synapses in the upper cortical layers. Next, we investigated the level of spontaneous activity of CRN and how they functionally integrate into early immature cortical networks using calcium imaging in organotypic cultures (OTCs), and immunohistochemistry in acute slices ex vivo. To characterize the chloride transporter expression in CRN at different developmental stages, we performed multiplexed fluorescence in situ hybridization (FISH). We then assessed the effect of pharmacological inhibition of NKCC1 on the survival of ΔNp73-positive CRN in OTCs. Finally, to evaluate the functional consequences of CRN activation, we employed two distinct optogenetic protocols. In the acute paradigm, we combined optogenetic stimulation of CRN with microelectrode array (MEA) recordings to determine the effect of CRN activation on early cortical network activity. In a separate paradigm, we recurred to chronic activation of CRN at later developmental stages to examine whether sustained activity affects their developmental loss.

## Materials and methods

### Animals

All animal experiments were conducted in accordance with the National and European (86/609/EEC) directive for the use of animals in research and the NIH Guide for the Care and Use of Laboratory Animals, and approved by the local ethical committee (Landesuntersuchungsanstalt RLP, Koblenz, Germany). All efforts were made to minimize the number of animals and their suffering. ΔNp73Cre^IRESGFP^ (ΔNp73Cre) [13] and Ai14 line (Strain# 007914) B6.Cg-Gt(ROSA)26Sor^TM14(CAG−tdTomato)Hze/J^ [33] transgenic mice were kept in a C57BL/6J background. ΔNp73Cre line was crossed with Ai14 reporter line to permanently label CRN. BaxCKOΔNp73Cre lineage [15, 16] was maintained on a mixed B6;129 genetic background. BaxCKOΔNp73Cre refers to the crossbreeding of ΔNp73Cre with Bax^tm2Sjk^;Bak1^tm1Thsn/J^ [34] and with Ai14 reporter line to conditionally inactivate *Bax* function in ΔNp73Cre-positive CRN and to permanently label them. All animals were housed in the local animal facility. Mice from postnatal day (P) 0 (day of birth) to P14 were sacrificed by decapitation; animals older than 2 weeks were deeply anesthetized with isoflurane (CP-Pharma; Cat# 1214) before being killed by cervical dislocation.

### Organotypic neocortical slice cultures

Organotypic neocortical slice cultures (OTCs) were prepared from P0-3 mice as previously described [35]. After decapitation, the brain was quickly removed and transferred into ice-cold Minimal Essential Medium (MEM, Gibco; Cat# 51200-046) supplemented with 2 mM Glutamax (Gibco; Cat# 35050-038), 4.5 mg/ml of glucose, 25 mM HEPES buffer solution (Gibco; Cat# 15630-056), 100 units/ml of penicillin and streptomycin mix, 2 mM L-Glutamine (Gibco; Cat# 25030-081) and pH adjusted to 7.3–7.4. Coronal brain slices (300 μm thick) were cut on a vibratome (Campden Instruments Ltd.) and the dorsolateral part of the neocortex was microdissected from the brain slices after pia removal. Isolated cortical slices were transferred onto Millicell cell culture insert (0.4 μm pore size, Millipore; Cat# PICM0RG50) and placed into a 35 mm petri dish. OTCs were kept at 37 °C, 5% CO_2_ in a culture medium with the following composition: L-Glutamine-enriched MEM (Gibco; Cat# 21575-022) supplemented with 25% horse serum and HBSS^+/+^ (Gibco; Cat# 24020-091), 1 mM Glutamax, 6 mg/ml of glucose, 10 units/ml of penicillin and streptomycin (Sigma-Aldrich, Merck), and pH adjusted to 7.2. Culture medium was renewed the day after preparation and subsequently every 2–3 days. For pharmacologically treated cultured slices, bumetanide (Tocris; Cat# 3108) was prepared as 1 mM stock solution in DMSO solution and diluted 1:100 into the culture medium to reach a final concentration of 10 µM. Bumetanide was applied from day in vitro (DIV) 1 on and replenished with every medium change. For control condition, equal volumes of solvents were applied.

### Viral transduction of organotypic neocortical slice cultures

Recombinant AAV (rAAV) carrying the following pAAV transfer vectors were used to transduce OTCs at DIV0: pAAV-EF1a-double floxed-hChR2(H134R)-EYFP-WPRE-HGHpA (Addgene; Cat# 20298-AAV9) to express the Cre-dependent channelrhodopsin-2 H134R mutant, pAAV.Syn.GCaMP6s.WPRE.SV40 (Addgene; Cat# 100843-AAV9) to express the GCaMP6s calcium indicator under the pan-neuronal Synapsin promoter, and pAAV.CAG.Flex.NES-jRCaMP1b.WPRE.SV40 (Addgene; Cat# 100849-AAV1) to express the Cre-dependent jRCaMP1b calcium indicator under the ubiquitous CAG promoter with cytosolic localization conferred by the Nuclear Export Signal (NES) sequence. Titer concentrations ranged from 7 × 10^12^ to ≥ 1 × 10^13^ vector genomes/ml and viral volumes between 0.5 and 2 µl were delivered directly onto OTC surface or added to the culture medium.

### Acute slice preparation

For preparation of acute brain slices, the brain was immediately removed after decapitation and placed in ice-cold artificial cerebrospinal fluid (ACSF), oxygenated with 95% O_2_ and 5% CO_2_, with the composition (in mM) 125 NaCl, 25 NaHCO_3_, 1.25 NaH_2_PO_5_, 1 MgCl_2_, 2 CaCl_2_, 2.5 KCl, 10 glucose (pH 7.4, 306 mOsm). Coronal sections of 350–400 μm thickness including the somatosensory cortex were cut on a vibratome (Campden Instruments Ltd.). Slices for functional analysis with MEA recordings were subsequently equilibrated for 20 min in oxygenated ACSF enriched with choline chloride (37.5 mM) at 32.5° C, before incubation in either control ACSF or ACSF supplemented with 20 µM 4-Aminopyridine (4-AP, Sigma-Aldrich; Cat# A78403-25G), both at 32.5 °C equilibrated with 95% O_2_ and 5% CO_2_ prior to electrophysiological recording or fixation for immunohistochemistry. Slices for immunohistochemical analyses of GABAergic synapse distribution were equilibrated for 1 h at room temperature in an oxygenated ACSF-filled chamber prior to fixation, as done previously [36].

### Immunohistochemistry

For immunohistochemical analysis, specimens (OTCs, acute brain slices or brain samples) were fixed in 4% formaldehyde in phosphate buffer (ROTI^®^ Histofix 4%, Carl Roth; Cat# P087.4) for at least 30 min at room temperature or overnight at 4° C and then washed with 0.01 M phosphate buffer solution (PBS). After cryoprotection with 30% sucrose solution in PBS, coronal brain sections of 100 μm thickness were cut from brain samples on a cryostat. After fixation, samples were stored in PBS at 4 °C until further use. Unspecific binding of antibodies was blocked and cells were permeabilized with 7% normal donkey serum (Jackson ImmunoResearch; Cat# 017-000-121), 0.8% triton (Triton^®^ X-100, Sigma-Aldrich; Cat# T8787) and 0.05% sodium azide (Sigma-Aldrich; Cat# S2002) in PBS 0.01 M for 2 h at RT or overnight at 4 °C. Afterwards, slices were incubated for about 65 h at 4 °C in 0.01 M PBS with 2% bovine serum albumin (Jackson ImmunoResearch; Cat# 001-000-161), 0.05% sodium azide, 0.3% triton, and one of the following primary antibodies: guinea pig polyclonal anti-vGAT (1:500, Synaptic Systems; Cat# 131004), rabbit recombinant c-Fos (1:1000, Synaptic Systems; Cat# 226008), goat polyclonal anti-GFP (1:200, Sicgen; Cat# AB0020-200). After washing in PBS, DAPI (AppliChem; Cat# A4099.0005) and at least one of the following fluorophore-conjugated secondary antibodies was used: Alexa Fluor 647-conjugated AffiniPure F(ab)2 donkey anti-guinea pig (1:200, Jackson ImmunoResearch; Cat# 706-606-148), Alexa Fluor 647-conjugated AffiniPure donkey anti-rabbit (1:200, Jackson ImmunoResearch; Cat #711-605-152) or Alexa Fluor 488-conjugated AffiniPure donkey anti-goat (1:200, Jackson ImmunoResearch; Cat# 705-545-147). Slices were subsequently mounted in Fluoromount-G (Biozol; Cat# SBA-0100-01).

### Multiplexed fluorescence in situ hybridization (FISH)

For FISH analysis, brains were removed and fixed overnight at 4° C in 4% formaldehyde in PBS. Afterwards, they were transferred to 30% sucrose solution in PBS for cryoprotection and 30 μm coronal slices were cut on a cryostat. Thereafter, slices were stored in PBS with diethylpyrocarbonate (DEPC) to ensure RNA integrity until hybridization was performed. Multiplexed FISH experiments were performed using the ViewRNA ISH tissue kit (Invitrogen; Cat# QVT0700). The following commercially available probes were used for RNA detection: *Slc12a5* (KCC2, Type 4, Invitrogen; Cat# VB4-3112562-VC) and *Slc12a2* (NKCC1, Type 6, Invitrogen; Cat# VB6-19636-VC). The protocol was performed according to the manufacturer’s guidelines with slight adaptations, as previously described [37]. Slices were mounted on Superfrost Plus™ Gold Adhesion Microscope Slides (Epredia; Cat# K5800AMNZ72) and heat-treated at 60 °C for 15 min before a hydrophobic barrier was drawn around the samples in order to keep the reagents in contact with the samples. Afterwards, slices were permeabilized using protease QF diluted 1:500 in pre-warmed PBS with DEPC (DPBS) at 40 °C for 5 min, followed by a second fixation with 4% paraformaldehyde (PFA) for 5 min at RT. Samples were thoroughly washed with DPBS before hybridization of the target probes. All the following incubation steps were done at 40 °C followed by three washing steps with DPBS or wash buffer for 2 min, if not indicated otherwise. The probes were diluted 1:40 in the probe set diluent QF and the incubation lasted for 2 h. Afterwards, the slices underwent two 30 min-incubation steps with the working PreAmplifier mix solution and then with the Amplifier mix solution. Both solutions were prepared by diluting 1:25 either the PreAmplifier mix or the Amplifier mix in pre-warmed amplifier diluent QF. After the signal was amplified, the samples were treated with the label probe mix diluted 1:25 in pre-warmed label probe diluent QF. Here, KCC2 was labeled with FITC and NKCC1 with Cy5 fluorochrome. In the end, slices underwent a final washing step with the last wash lasting 10 min. Afterward, slices were mounted with Immunoselect Antifading Mounting Medium with DAPI (DIANOVA; Cat# SCR-038488).

### Imaging

Epifluorescence images were taken with 4x (UPlanSApo 4x/0.16 NA, Olympus) and 10x (UPlanFLN 10x/0.30 NA, Olympus) objectives on an Olympus IX81 epifluorescence microscope connected to a CCD camera (XM10, Olympus) using the cellSens software (Olympus). Fluorescence excitation was provided by a Lumencor SOLA SE II light source and excitation/emission bandpass selection was performed using the following Olympus filter cubes: U-MNU2 (Ex 360–370 nm; Em > 420 nm, long-pass filter), U-N41001 (Ex 480/40 nm; Em 535/35 nm), U-N41002 (Ex 535/35 nm; Em 610/75 nm), and U-N41033 (Ex 640/30 nm; Em 700/75 nm). When higher magnification and z-stack acquisition were required, images were acquired using a spinning disk confocal system (5 Elements, Visitron), consisting of a Yokogawa spinning disk head (CSU-W1, 50 μm pinholes) attached to a Nikon Ti2-E microscope body equipped with a 60× water immersion objective (CFI Plan Apo VC 60XWI, NA 1.20, Nikon) and a Prime BSI sCMOS camera (2048 × 2048 pixels, 6.5 μm pixel size, Photometrics) controlled by VisiView software (Visitron). Fluorescence excitation was conducted at 405 nm, 488 nm, 561 nm and 640 nm using diode lasers emitting at 405 nm/488 nm/640 nm (Toptica) and a diode pumped laser (DPL) at 561 nm (Cobolt). Emitted fluorescence was filtered by bandpass filters 460/50 (Chroma ET460/50) for 405 nm excitation, 525/50 (Chroma ET525/50) for 488 nm excitation, 623/32 (Semrock FF01-623/32) for 561 nm excitation, and a 633 long-pass filter (Semrock BLP01-633R) for 640 nm excitation. Z-stacks were acquired using a z-step of 0.5–1.0 μm, depending on the experimental requirements.

### Image analysis

After images acquisition, analyses were conducted in Fiji ImageJ [38] and Imaris 10.0 (Oxford instruments) software. For GABAergic synaptic profiling of CRN, dendrites were reconstructed with Filament Tracer function of Imaris, while vGAT profiles were modeled using the Spot function with spots radii of 0.5 μm. Putative GABAergic synapses, represented by the presynaptic component vGAT, were assigned to CRN dendrites when located within an absolute distance of maximum 1.33 μm, adapting a previously described method [36]. For CRN and vGAT counts, their absolute numbers were normalized to the volume of the field of view (FOV). For the characterization of CRN activation in acute slices, c-Fos signal in upper layers was quantified from epifluorescence images using Fiji software. Region of interests (ROIs) within the somatosensory cortex were manually defined, and the background-corrected intensity was calculated by subtracting the ROI’s minimum grey value from the ROI’s mean grey value. c-Fos-positive CRN were quantified from confocal z-stacks by identifying CRN somas exhibiting colocalized nuclear c-Fos immunoreactivity. For FISH analysis, Fiji software was used to analyze confocal z-stack images. Only isolated, non-overlapping cells were included, and one single optical plane corresponding to the center of each cell was analyzed. Each punctum corresponded to a single RNA particle. Puncta located in the somatic area with an area 20% larger than DAPI-identified nucleus were assigned to individual cells, and for each probe the number of puncta/cell was quantified. CRN density of OTCs over time was quantified with a custom-written macro in Fiji. In brief, 4x epifluorescence images of tdTomato^+^ CRN were stitched together with Grid/Collection stitching plugin [39]. Afterwards, a background correction using a rolling ball algorithm with a size of 50 pixel was applied, followed by the Threshold function to generate binary masks. These were refined using the “Watershed” function to separate touching or overlapping particles. Subsequently, the function “Analyze Particles” with a minimum particle size of 15 pixel and a maximum size of 175 was applied to only count particles reflecting the size of CRN somata. Cre-specific ChR2(H134R)-EYFP transduction in OTCs was assessed based on GFP signal in epifluorescence images in Fiji. tdTomato^+^ CRN and GFP^+^ cells were manually counted for each FOV and transduced CRN were quantified based on somatic colocalization of signals. AAV specificity was calculated as number of transduced CRN / total number of transduced cells while efficiency of transduction was calculated as transduced CRN / total number of tdTomato^+^ CRN.

### Multi electrode array (MEA)

Extracellular electrophysiological recordings were performed using planar 120-channel microelectrode arrays (120MEA200/30iR-Ti-pr, Multi Channel Systems) for organotypic cortical cultures and three-dimensional microelectrode arrays (120-3DMEA250/12/100iR-Ti-pr, Multi Channel Systems) for acute slices with a MEA2100 system (Multi Channel Systems). Electrodes had a layout of 12 × 12 with a diameter of 30 μm and an inter-electrode (center to center) distance of 200 μm for planar MEAs, and diameter of 12 μm, an inter-electrode distance of 250 μm and an electrode height of 100 μm for 3-D MEAs. The day before recordings, MEAs were coated with a polyornithine solution diluted 1:500 in HBSS^−/−^ (Gibco; Cat# 14175-053) to promote tissue adhesion. Acute cortical slices were placed on the MEA, stabilized on the electrodes with a nylon grid and constantly perfused with oxygenated ACSF at 32 °C with a temperature controller (TC02, Multi Channel Systems). OTCs were perfused with a low-Mg^2+^ ACSF, with MgCl_2_ adjusted to 0.5 mM to promote network activity. Slices and OTCs were incubated 5–10 min on the MEA chips before start of recordings to ensure stable contact with the electrodes. Analog signals were recorded with Multi Channel Experimenter 2.2 software (Multi Channel Systems) at a sampling rate of 10 kHz for 10 min per recordings. Spontaneous local field potentials (LFPs) of acute slices were analyzed using the Multi Channel Analyzer 2.2 software (Multi Channel Systems). Raw traces were low-pass filtered at 20 Hz (2nd-order Butterworth) and events were detected with an automatic threshold-based detector set to 6x standard deviation of the noise level, with 1 s dead time between the detected events. Electrodes were considered active if > 2 LFPs were detected during 10 min recordings. To calculate the event rate, the 20% most active electrodes were included when ≥ 50 electrodes were active; otherwise, all active electrodes were included. Conversely, raw MEA recordings from OTCs were exported in HDF5 format and analyzed with a custom-made Python script. In brief, traces were downsampled to 100 Hz and then low-pass filtered using a 2nd-order Butterworth filter with 20 Hz cut-off. Events larger than 7x standard deviation of the noise level were collected. Dead time of 0.8 s between detected events was applied and when optogenetic light trains were delivered during MEA recordings, burst periods were excluded from the calculation of spontaneous event rate. Active electrodes were defined if they exhibited > 2 spontaneous events during the recording. For each sample, only the electrodes that were considered active during the spontaneous MEA recording without optogenetic stimulation were considered for the event rate calculations of MEA recording with optogenetic stimulation. The amplitude of spontaneous network events was calculated as the maximum voltage deflection from baseline within 500 ms upon threshold passing. For each spontaneous event detected with and without repeated burst stimulation, the number of electrodes involved was quantified. To characterize event spread, events involving less than 5 electrodes were defined as local non-spreading events, while events involving more than 10 electrodes were defined as global spreading events. The relative number of electrodes involved in local and global spreading events was calculated as the number of participating electrodes relative to the total number of active electrodes per recording. Optogenetically evoked intraburst events were quantified separately using the same workflow; light artifacts were excluded by calculating dynamically for each electrode the noise standard deviation from the 0.5 s baseline preceding the first pulse, and no dead time was applied. Evoked response reliability was calculated as follows: (total number of evoked responses / total number of stimuli) per electrode.

### Optogenetic light stimulation

Blue light (470 nm) was applied to OTCs from high-power LED (Roschwege; Cat# XPEBBL-L1-0000-00201) with an average light intensity of approximately 8–9 mW/mm^2^ mounted on a heat-dissipating metallic support. Stimulation typically consisted of a train of 10 light pulses with a pulse duration of 3 or 10 ms at a frequency of 20 Hz every 10 or 20 s. LED stimulation was controlled by a custom-built Arduino Uno Rev3 (Arduino) microcontroller board based on the ATmega328P. For stimulation of OTCs during simultaneous MEA-based electrical recordings, a single LED circuit was built and positioned under the head stage of the MEA recording system, and the on and off timing of the LED was registered as digital input to the MEA amplifier. For the chronic light stimulation of OTCs, a custom-designed 12-well LED array controlled by an Arduino was used [40]. The LED array was positioned in the incubator, maintaining physiological conditions and thus allowing for long-term stimulation. During chronic stimulation, each OTC insert was transferred into an-individual 35 mm Petri dish containing culture medium allowing a dedicated LED to be positioned beneath each dish.

### Calcium imaging

Ca^2+^ imaging was carried out with an upright microscope (BX61WI, Olympus) connected to a digital CCD camera (ORCA-R2, C10600-10B, Hamamatsu Photonics K.K.) and a xenon arc light source (MT20-E, Olympus). OTCs were transferred to a recording chamber and secured with a nylon grid. Cultures were constantly perfused with low-Mg^2+^ oxygenated ACSF (MgCl_2_ 0.5 mM) and maintained at 32 °C. Fluorescence images were taken with a 10× water-immersion objective (UMPLFLN10XW, Olympus) and acquired with xcellence software (Olympus). Time-lapse videos were acquired at 5 Hz frequency per channel with alternating FITC (Ex 485/20 nm; Em 521 nm) and Cy3 (Ex 560/25 nm; Em 607 nm) filter sets. Each recording lasted 10 min. Image preprocessing was performed in Fiji software. Maximum intensity projections of time-lapse recordings were generated, and ROIs were manually drawn to delineate individual neurons, while large-area ROIs were defined as a proxy for network-wide activity. Raw calcium traces were extracted by computing ROIs mean gray values for every frame using the Multi Measure function in Fiji and analyzed with a custom-made Python script partially adapted from previously published approaches [41]. Raw traces were corrected by subtracting background intensity and converted to ΔF/F_0_ using a robust baseline F_0_(t), computed as the mean of the 10th-20th percentiles of the signal intensity within a centered rolling window (30 s). For recordings of jRCaMP1b fluorescence, noise level (σ) was estimated from a ΔF/F_0_ Gaussian fit to the left side of the histogram. Conversely, for GCaMP6s fluorescence σ was calculated as the standard deviation of the lowest 20% of ΔF/F_0_ values across the entire recording. Afterwards, ΔF/F_0_ traces were corrected by subtracting the computed noise mean. Calcium transients were detected using a combination of static threshold (3σ for jRCaMP1b and 5σ for GCaMP6s recordings) and dynamic threshold (95% confidence interval). Active periods were defined when ΔF/F_0_ values were above the threshold for ≥ 2 s. To prevent baseline drift during prolonged activity time, the rolling F_0_(t) was frozen when ΔF/F_0_ remained above threshold for ≥ 5 s, and maintained for an additional 2 s after event termination. Threshold was subsequently reapplied to the updated ΔF/F_0_ traces. ROIs were classified as active when ≥ 2 active time windows were detected. ΔF/F_0_ traces were binarized into active/inactive frames, and pairwise co-activity overlap between single neurons was quantified using the Jaccard index, defined as the number of co-active frames (intersection time) divided by the number of frames in which at least one cell was active (union time).

### Statistics

Values are reported as mean ± SEM. All statistical tests were performed using GraphPad Prism 10 (GraphPad). Normality of sample distributions was tested with Shapiro-Wilk test and a Bartlett’s test was applied to test for homoscedasticity. Parametric tests were applied when normality was not rejected in ≥ 80% of groups and their variances were homogeneous (for unpaired datasets) or when model residuals met these assumptions (for paired datasets); otherwise, appropriate non-parametric tests were used. Accordingly, comparisons between multiple groups were made either with one-way ANOVA or a Kruskal-Wallis test. Two-way repeated measures ANOVA was used to compare multiple groups with different factors followed by multiple comparison tests (Tukey’s or Šidák’s). Parametric comparisons between two groups were made with unpaired or paired Student’s t-test, while nonparametric Mann-Whitney or Wilcoxon tests were applied, when appropriate. Significance was considered at *p* values < 0.05 and significant differences were indicated by asterisk *p < 0.05; **p < 0.01; ***p < 0.001; ****p < 0.0001.

## Results

### Neocortical CRN display high GABAergic input density before their disappearance

**Fig. 1:**
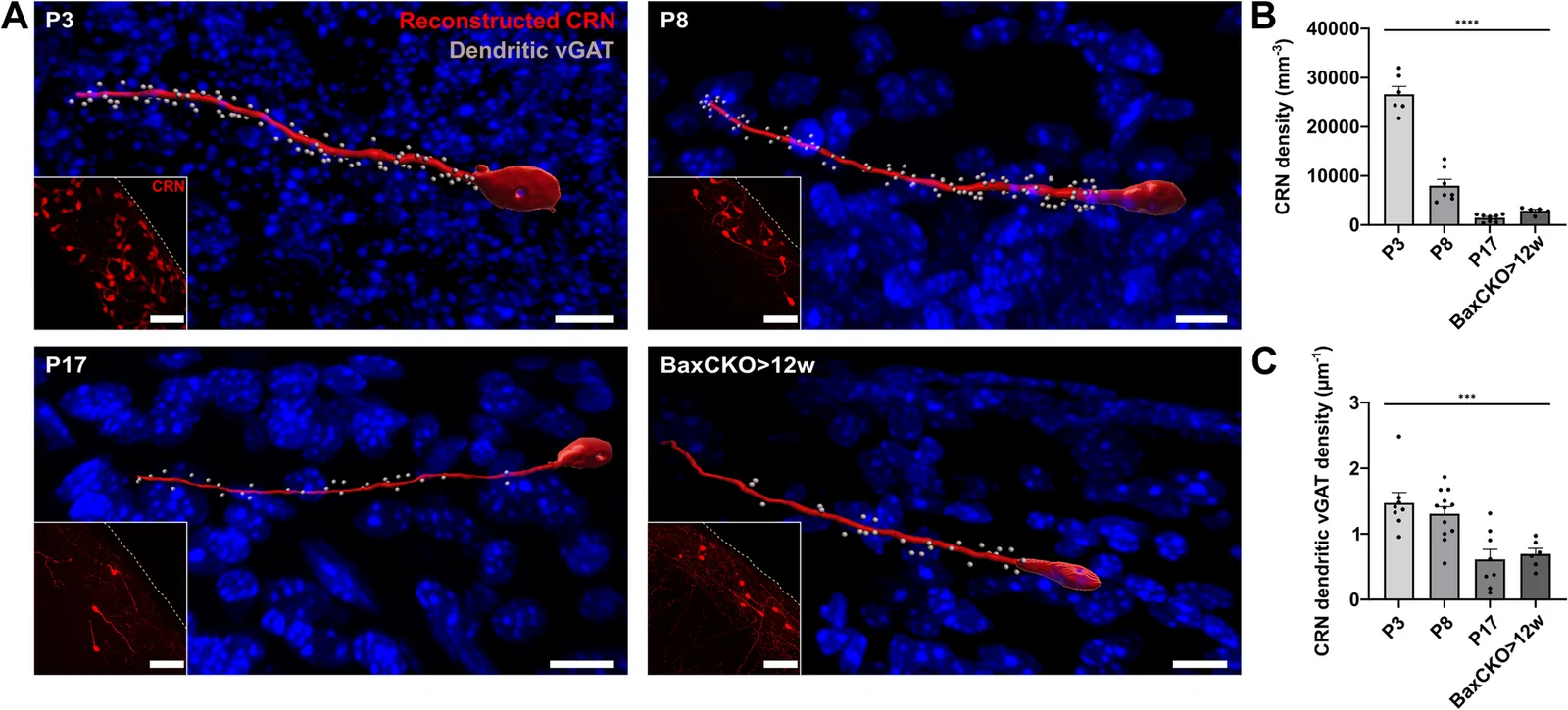
Quantification of CRN density and CRN dendritic vGAT density in upper neocortical layers throughout development. **A** Representative reconstructions of single ∆Np73^+^ Cajal-Retzius neurons (CRN, red) with vGAT-positive puncta attributed to their dendrites (gray) alongside representative images of tdTomato^+^CRN in the upper cortical layers (insets) from murine coronal slices at P3, P8, P17 and adulthood (> 12 weeks using the BaxCKO∆Np73Cre^+^ mouse line). Scale bars: overview = 10 μm, insets = 50 μm. **B**, **C** Quantification of CRN density in upper layers and dendritic vGAT density on CRN from P3 to adulthood. The number of CRN sharply decreased from P3 to P8, and decreased further at P17. In adult BaxCKO∆Np73Cre^+^ mice, however, suppression of the expression of the pro-apoptotic factor BAX preserved a subpopulation of CRN. The density of vGAT-positive pucta along the dendrite of CRN also decreased across development, with the sharpest reduction occurring between P8 and P17. Meanwhile, between P3 and P8 the density remained similar. For quantification of CRN density: *n* = 6 (P3), 7 (P8), 8 (P17), 5 (BaxCKO>12w) FOV from at least 4 slices per group. For quantification of dendritic vGAT density: *n* = 8 (P3), 12 (P8), 8 (P17), 6 (BaxCKO>12w) cells from at least 4 slices per group. ***p < 0.001, ****p < 0.0001 (Kruskal-Wallis test)

During the first week after birth Cajal-Retzius neurons (CRN) represent one of the main targets of GABAergic synapses in the marginal zone of mice [21, 23, 25, 30, 36], with most of these GABAergic inputs presumably originating from layer I (LI) interneurons [19, 20]. To investigate how GABAergic presynaptic innervation onto CRN changes during cortical development and CRN lifespan, we longitudinally assessed GABAergic synaptic inputs to CRN across development. Therefore, we performed immunohistochemical staining against the presynaptic marker vGAT at postnatal days (P) 3, 8, 17 and in adulthood (> 12 weeks) in ΔNp73Cre^+^ mice [13] in which CRN were genetically labelled by crossing with reporter Ai14 mice. In these mice, tdTomato reporter was expressed in the vast majority of hem- and septum-derived cortical CRN (Fig. 1A).

Because of the transient nature of neocortical CRN, GABAergic characterization of adult CRN was performed when death of ΔNp73-positive CRN was impaired through additional, Cre-specific inactivation of the pro-apoptotic factor *Bax* (BaxCKOΔNp73Cre lineage). The results (Fig. 1B) showed that, as expected, CRN density in the upper cortical layers of the somatosensory cortex decreased significantly (Kruskal-Wallis test, *p* < 0.0001) with postnatal age from 26.6 ± 1.7 × 10^3^ mm^− 3^ at P3, 79.9 ± 1.3 × 10^3^ mm^− 3^ at P8, to 14.6 ± 2.7 × 10^3^ mm^− 3^ at P17. In BaxCKOΔNp73Cre mice older than 12 weeks, when *Bax*-dependent apoptosis is inhibited and survival of a subpopulation of CRN is increased [15], CRN density was preserved at 28.8 ± 3.1 × 10^3^ mm^− 3^. Next, we identified putative GABAergic inputs to CRN. To this end, we first determined the dendritic surface of tdTomato^+^ CRN as well as the profile of vGAT signals. Adapting a previously described method [36] we then identified putative GABAergic synapses onto CRN as vGAT-positive spots in close proximity (1.33 μm or less) to dendritic surface of a tdTomato^+^ CRN (Fig. 1A, Supplementary Fig. 1A). The results showed that CRN dendritic vGAT density significantly (Kruskal-Wallis test, *p* = 0.0004) changed with age, with a slight decrease from 1.5 ± 0.2 μm^− 1^ at P3 to 1.3 ± 0.1 μm^− 1^ at P8, before it markedly declined to 0.6 ± 0.2 μm^− 1^ at P17. In adult *Bax* conditional knock-out mice, persistent CRN displayed a vGAT dendritic density, which was similar to that in P17 naïve mice (Fig. 1C). To examine the global GABAergic synaptogenesis in the upper cortical layers, we also performed longitudinal quantification of vGAT density with postnatal age. In line with previous findings [36, 42, 43], vGAT density increased in a significant way from P3 to P8 before stabilizing to at P17, and 12 weeks or more (Supplementary Fig. 1B). In conclusion, GABAergic synaptic inputs to CRN, quantified by dendritic vGAT density, decreased as CRN became less abundant across development. Conversely, overall vGAT density in upper layers increased with age, consistent with the maturation of GABAergic synaptic connectivity during development.

### CRN are spontaneously active and functionally integrated into cortical networks at early development

**Fig. 2:**
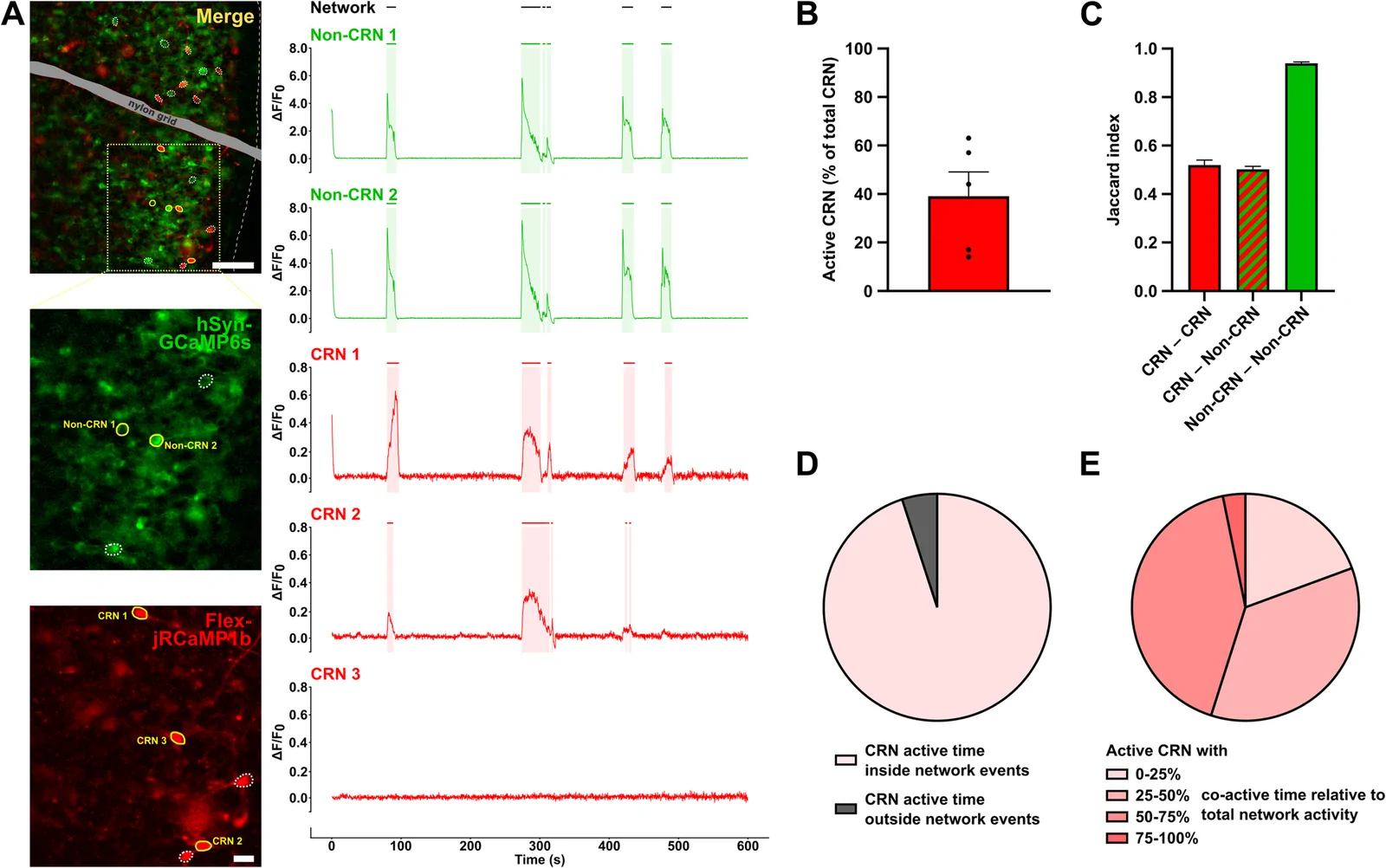
Calcium imaging-based characterization of CRN and non-CRN activity in cortical organotypic cultures.** A** Representative maximum-intensity time projections of calcium signals (left) and corresponding single-cell ΔF/F_0_ traces as well as network active time periods (right) from two non-CRN and three CRN in a cortical organotypic culture (OTC) at DIV8. The OTC was previously transduced with AAV encoding the non-specific calcium indicator GCaMP6s (green, for non-CRN) and the Cre-specific jRCaMP1b (red, for CRN). Scale bars = 100 μm. **B** Quantification of the active proportion of CRN per OTC (active CRN / total jRCaMP1b-positive CRN). Approximately 40% of CRN across the OTCs were identified as active (≥ 2 active time periods lasting ≥ 2 s). **C** Quantification of binary co-activity overlap (Jaccard index) for CRN-CRN, CRN-non-CRN, and non-CRN-non-CRN pairs. CRN displayed a similar co-activity overlap within CRN group and with non-CRN, while non-CRN showed near complete overlap. **D** Characterization of temporal overlap between CRN activity and network events. Over 90% of CRN activity occurred during network events. **E** Distribution of relative co-active time of active CRN with network-wide events. Most active CRN (~ 80%) had a relative active time that covered between 25 to 75% of the cortical network’s total active time. *n* = 86 (CRN), 41 (non-CRN neurons) from 5 OTCs. For analysis of co-activity: *n* = 109 (CRN-CRN pairs), 256 (CRN-non-CRN pairs), 148 (non-CRN-non-CRN pairs)

While we know that CRN in the postnatal cortex display relatively mature electrophysiological properties, their functional connectivity into maturing cortical networks is still poorly understood [2, 44]. Dense GABAergic presynaptic inputs to CRN from birth on, reinforce the question of whether CRN are functionally integrated into early neocortical networks and to what extend CRN activity correlates with the stereotypic network-wide activity patterns typically present in immature neocortical networks [45, 46]. To address these questions, we focused on the first postnatal week, when CRN are highly abundant in layer I and before the onset of their natural cell death. In order to simultaneously record activity in CRN and the general network activity, organotypic cultures (OTCs) were co-transduced with recombinant adeno-associated viruses (AAVs) carrying two genetically encoded Ca^2+^ indicators (GECIs): red fluorescent Cre-dependent jRCaMP1b to specifically report calcium activity of ΔNp73Cre^+^ CRN, and green fluorescent GCaMP6s under the pan-neuronal Synapsin promoter to report pan-neuronal calcium activity. By this means, dual channel calcium imaging was then performed at DIV7 or 8 in order to simultaneously record network activity states as well as single-cell calcium dynamics in both CRN (identified by jRCaMP1b expression and the typical CRN shape) and non-CRN neurons (GCAMP6s-positive, jRCaMP1b-negative). For comparison, network activity state was assessed from large ROIs in lower layers of the OTCs. In order to obtain a sufficient number of network events during the limited recording interval, we increased the frequency of recurrent network oscillations in immature OTCs [45, 47] using low-magnesium recording conditions [48]. As a result, OTCs at DIV7/8 showed robust network-driven population activity (0.4 ± 0.1 events/min). Both GECIs reliably reported the activity of CRN and non-CRN neurons, respectively (Fig. 2A). As expected, activity of non-CRN neurons occurred in almost complete synchrony with the registered network state. However, calcium activity appeared to be slightly less frequent in CRN. Quantification of the relative fraction of spontaneously active CRN revealed that 39 ± 10% of jRCaMP1b-transduced CRN were active (Fig. 2B).

Temporal co-activity was analyzed using the Jaccard index applied to binarized calcium traces for CRN-CRN, CRN-non-CRN, and non-CRN-non-CRN pairs. Active CRN displayed a similar co-activity overlap with other CRN (0.5 ± 0.02) as compared with non-CRN (0.5 ± 0.01). While non-CRN cells showed near-complete temporal overlap (0.9 ± 0.01; Fig. 2C), which is in agreement with the high synchrony and network-wide extent of early oscillatory activity in immature cortical networks [49]. Moreover, characterization of the temporal overlap of CRN activity with network event revealed that 95 ± 2% of CRN active time coincided with network-wide activity. Complementary, only 5 ± 2% of this CRN activity happened outside of network-wide events (Fig. 2D). To further examine CRN-network coupling, we calculated the relative co-active time of CRN with network-wide events and compared the relative distribution of this normalized co-activation across active CRN. Out of 31 spontaneously active CRN, the relative active time of 6 CRN (19.4%) overlapped with < 25% of the total network active time. 11 CRN (35.5%) had a relative co-active time of 25–50%, 13 CRN (41.9%) of 50–75% and only 1 CRN (3.2%) showed activity over more than 75% of network-wide activity time bins (Fig. 2E). Thus, these findings indicated that a substantial portion of CRN (around 40%) showed spontaneous calcium activity in OTCs. At single-cell level, CRN activity robustly correlated with neuronal calcium dynamics of both other CRN and non-CRN cells. Moreover, CRN active periods were temporally aligned with network-wide events and, for most active CRN, accounted for a large fraction of network’s total active time.

**Fig. 3:**
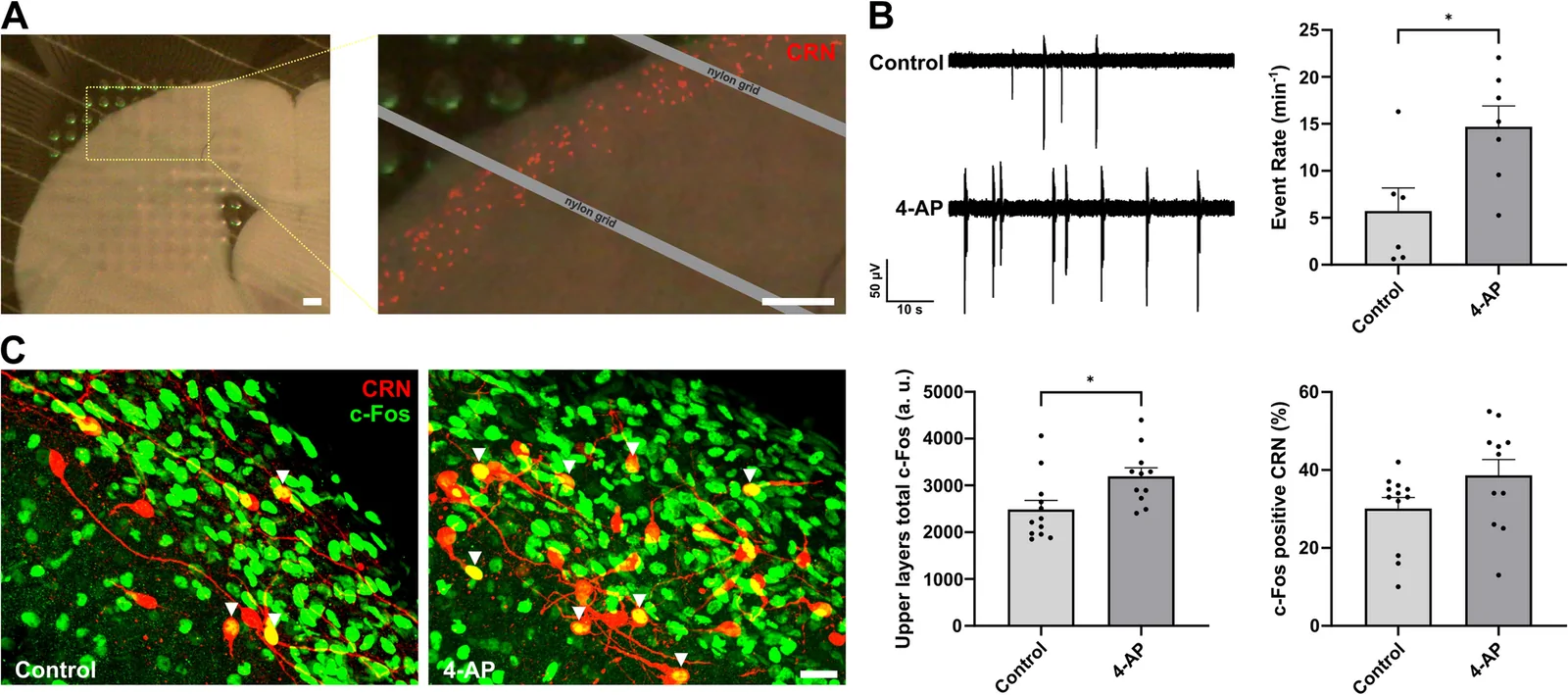
Electrophysiological and immunohistochemical assessment of neuronal activity and early CRN activation in ex vivo slices.** A** Representative images of a P8 acute slice on a 3D-multi-electrode array with close-up of tdTomato^+^ CRN (red) in the somatosensory cortex. Scale bars = 200 μm. **B** Representative field potential traces (left) and quantification of event rates (right) from P7/8 acute slices perfused with either control artificial cerebrospinal fluid (ACSF) or 20 µM 4-aminopyridine (4-AP) ACSF. As expected, 4-AP induced a significantly higher rate of spontaneous events compared to control. **C** Representative images of c-Fos-stained slices incubated in control ACSF or with 4-AP, and quantification of c-Fos immunoreactivity in cortical upper layers as well as of relative proportion of c-Fos^+^ CRN (highlighted with white triangles). Consistent with the electrophysiology results, 4-AP significantly increased overall c-Fos activity. Under control conditions, approximately 30% of CRN can be considered active based on c-Fos positivity. Interestingly, this percentage increased slightly with 4-AP-induced network stimulation, despite the dense GABAergic innervation of the CRN. Scale bar = 20 μm. For MEA recordings: *n* = 6 (control), 7 (4-AP-treated) slices. For immunohistochemistry: *n* = 12 (control), 11 (4-AP-treated) slices. *p < 0.05 (unpaired Student’s t-test or Mann-Whitney test)

Next, we combined electrophysiological recordings and immunohistochemical analysis to study the neuronal activity of early neocortical CRN in ex vivo brain slices. Immunohistochemical stainings against the immediate early gene c-Fos, a classical marker for neuronal activity, were performed on acute slices from ΔNp73Cre^+^/tdTomato^+^ mice at P7/8. To assess the impact of altered cortical network excitability on CRN activity, acute slices were incubated in control artificial cerebrospinal fluid (ACSF) or ACSF supplemented with the K^+^ channel blocker 4-aminopyridine (4-AP, 20 µM), which in the present study is used to substantially enhance neuronal network activity [50]. In a subset of experiments extracellular electrophysiological recordings were performed to validate the 4-AP-induced increase in network excitability (Fig. 3A).

The electrophysiological experiments demonstrated, as expected, that the rate of spontaneous network events was significantly (unpaired Student’s t-test, *p* = 0.02) higher in 4-AP-treated slices (14.7 ± 2.2 events/min) as compared to control (5.7 ± 2.5 events/min; Fig. 3B). Accordingly, slices incubated with 4-AP showed significantly (Mann-Whitney test, *p* = 0.01) higher total c-Fos levels in the upper layers of the somatosensory cortex as compared to untreated slices (3200 ± 200 vs. 2500 ± 200 a.u.). With respect to CRN c-Fos immunoreactivity, 30 ± 3% of all CRN were c-Fos-positive under control conditions, while in 4-AP-treated acute slices this proportion was slightly higher with 39 ± 4% (Mann-Whitney test, *p* = 0.1; Fig. 3C).

Altogether, based on c-Fos immunostainings in acute slices and the occurrence of calcium events under low-Mg^2+^ conditions in OTCs, a considerable percentage of CRN (~ 30%) showed spontaneous activity. CRN activity was highly correlated with network-wide dynamics and enhanced network excitability promoted CRN activation rather than inhibition, despite their dense GABAergic inputs.

### CRN maintain higher NKCC1 and lower KCC2 mRNA expression across their lifespan, which contributes to CRN cell death

**Fig. 4:**
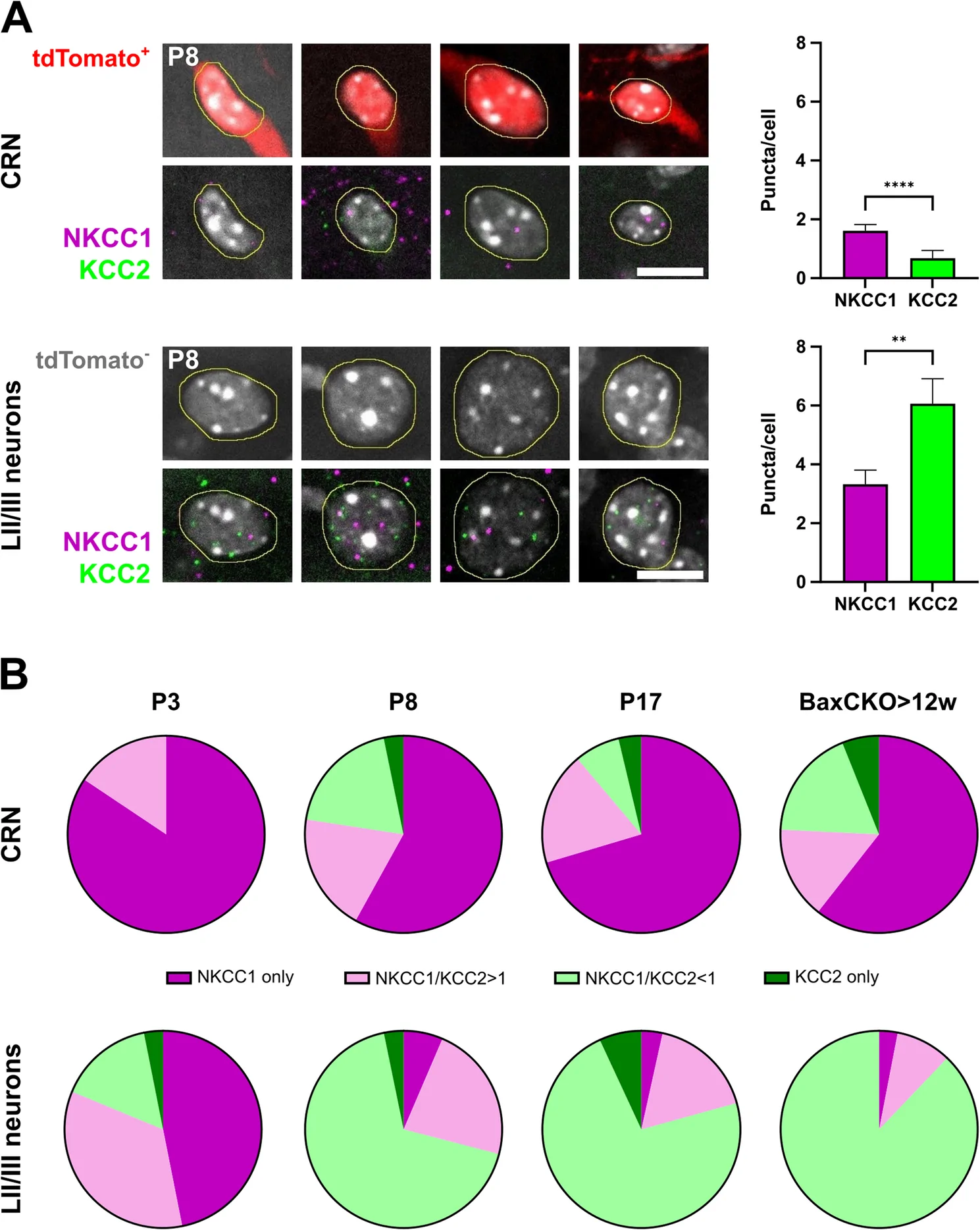
Longitudinal profiling of chloride transporter expression in CRN and layer II/III neurons across development.** A** Representative images of multiplexed FISH against NKCC1 (magenta) and KCC2 (green) in tdTomato^+^ CRN (red) and tdTomato^−^ layer II/III neurons at P8 (left) with quantification of single-plane puncta per cell shown on the right. CRN displayed low levels of KCC2 expression compared to layer II/III neurons. Scale bar = 10 μm. **B** Group-wise comparison of NKCC1/KCC2 ratios from P3 until adulthood. From early developmental stages, most CRN expressed more NKCC1 than KCC2 and maintained this profile throughout their entire lifetime. Similarly, in the BaxCKO∆Np73Cre^+^ lineage, CRN that survived past their natural developmental death maintained this particular expression profile into adulthood. In contrast, the expression profile of layer II/III neurons switched between P3 and P8, when KCC2 expression prevailed over NKCC1 expression until adulthood. *n* = 32 (P3), 31 (P8), 27 (P17), 32 (BaxCKO>12w) CRN; *n* = 32 (P3), 31 (P8), 29 (P17), 33 (BaxCKO>12w) LII/III neurons from 3 mice per time point. **p < 0.01, ****p < 0.0001 (paired Student’s t-test or Wilcoxon test)

Previous studies showed that GABA application elicited excitatory responses in CRN after birth and that chloride influx mediated by NKCC1 transporter was required to render GABA responses excitatory [51]. In addition, outward chloride transporter KCC2 mRNA was suggested to be absent in CRN during the second postnatal week [52]. Based on these findings, together with the developmental profile of CRN density, the vast GABAergic inputs to early CRN, and the functional connectivity of CRN presented above, we next compared the expression levels of the inward chloride transporter NKCC1 and of the outward chloride transporter KCC2 in CRN and in non-CRN cells across development. Therefore, our goal was to profile the mRNA expression of the main chloride transporters, NKCC1 and KCC2, throughout the development of CRN and layer II/III (LII/III) neurons. To do so, we performed multiplexed fluorescence in situ hybridization (FISH) against NKCC1 and KCC2 in brain slices from P3, P8 and P17 of ΔNp73Cre^+^/tdTomato^+^ mice, as well as from adult BaxCKOΔNp73Cre mice (> 12 weeks). As expected, LII/III neurons showed a prominent rise in KCC2 expression levels between P3 and P8 (from 1.1 ± 0.2 to 6.1 ± 0.8 puncta/cell) before expression levels increased again to adult levels from 5.5 ± 0.5 puncta/cell at P17 to 10.0 ± 1.5 puncta/cell in adult tissue. Overall, absolute expression levels for both transporters were generally higher in LII/III neurons compared to CRN (Supplementary Fig. 2). Comparison of the relative expression confirmed P8 as a key developmental time point, when the expression profile of chloride transporters in LII/III neurons switched to a significantly higher (paired Student’s t-test, *p* = 0.005) expression of KCC2 over NKCC1 (Fig. 4A). In contrast, CRN still showed significantly higher (Wilcoxon test, *p* < 0.0001) NKCC1 than KCC2 levels at P8. Group-wise comparison of the relative NKCC1/KCC2 expression from P3 until adulthood showed a consistently predominance of NKCC1 over KCC2 expression in CRN during postnatal development: 100% (32/32 CRN) at P3, 77% (24/31 CRN) at P8, and 89% (24/27 CRN) at P17, as well as 76% in late adulthood (25/32 CRN in adult mice older than 12 weeks). Conversely, layer II/III neurons displayed a prevalence of NKCC1-dominant neurons only at P3 (81% or 26/32 neurons). At P8 KCC2 mRNA levels already outnumbered NKCC1 and most of LII/III neurons displayed a predominance of KCC2 expression in 71% (22/31) of neurons. This KCC2-dominant expression profile was then maintained at P17 (79% or 23/29 neurons) and in adulthood (88% or 29/33 neurons; Fig. 4B). The particular expression profile of CRN reflected their consistently low expression levels of both NKCC1 and KCC2 across time. On contrary, absolute expression of NKCC1 steadily increased in layer II/III neurons yet being outnumbered by the stepwise increase in KCC2 expression mentioned earlier (Supplementary Fig. 2). The developmental expression shift forms the basis for the functional switch to mostly inhibitory GABAergic responses in layer II/III and other non-CRN neurons.

However, this functional maturation is precluded by the particular expression profile of chloride transporters in CRN throughout their lifespan, which is consistently characterized by predominance of NKCC1 expression.

Pharmacological blockade of NKCC1 was previously shown to alter survival rates of CRN in population-based investigations [35]. To investigate if NKCC1 function also affects survival of hem- and septum-derived ΔNp73-positive CRN in particular, we performed longitudinal cell-to-cell tracking of ΔNp73-positive CRN in cortical OTCs from DIV4 to DIV12. Survival of ΔNp73-positive CRN under chronic application of the loop diuretic bumetanide (10 µM), which is inhibiting the chloride transporter NKCC1 [53], was compared to control conditions (Supplementary Fig. 3). CRN density was normalized to DIV4-6 and amounted to 1 ± 0.02 at DIV7-9 (vs. 0.9 ± 0.04 in controls) and 0.9 ± 0.03 at DIV10-12 (vs. 0.8 ± 0.04). Hence, CRN density significantly decreased with time (2-Way RM ANOVA, Time *p* < 0.0001) in both groups. When CRN density at DIV7-9 and DIV10-12 was compared between bumetanide-treated and control conditions, bumetanide-treated cultures showed a trend toward higher density of surviving CRN (2-Way RM ANOVA, Experimental Groups *p* = 0.06, Interaction *p* = 0.06). In line with previous findings in the broader reelin-positive CRN population [35], this trend to increased survival rates in the presence of bumetanide suggests an association between blockade of NKCC1 by chronic bumetanide application in OTCs and a reduced death rate of ∆Np73^+^ CRN subpopulation.

Taken together, these findings showed that CRN present a particular chloride transporter expression profile throughout their lifespan with persistent predominance of NKCC1. While at early phases the resulting GABAergic excitation can mediate the positive network integration of CRN via prevalent GABAergic inputs, it can promote CRN death later on.

### Stimulation of CRN amplifies early neocortical network dynamics and accelerates CRN loss at later stages

**Fig. 5:**
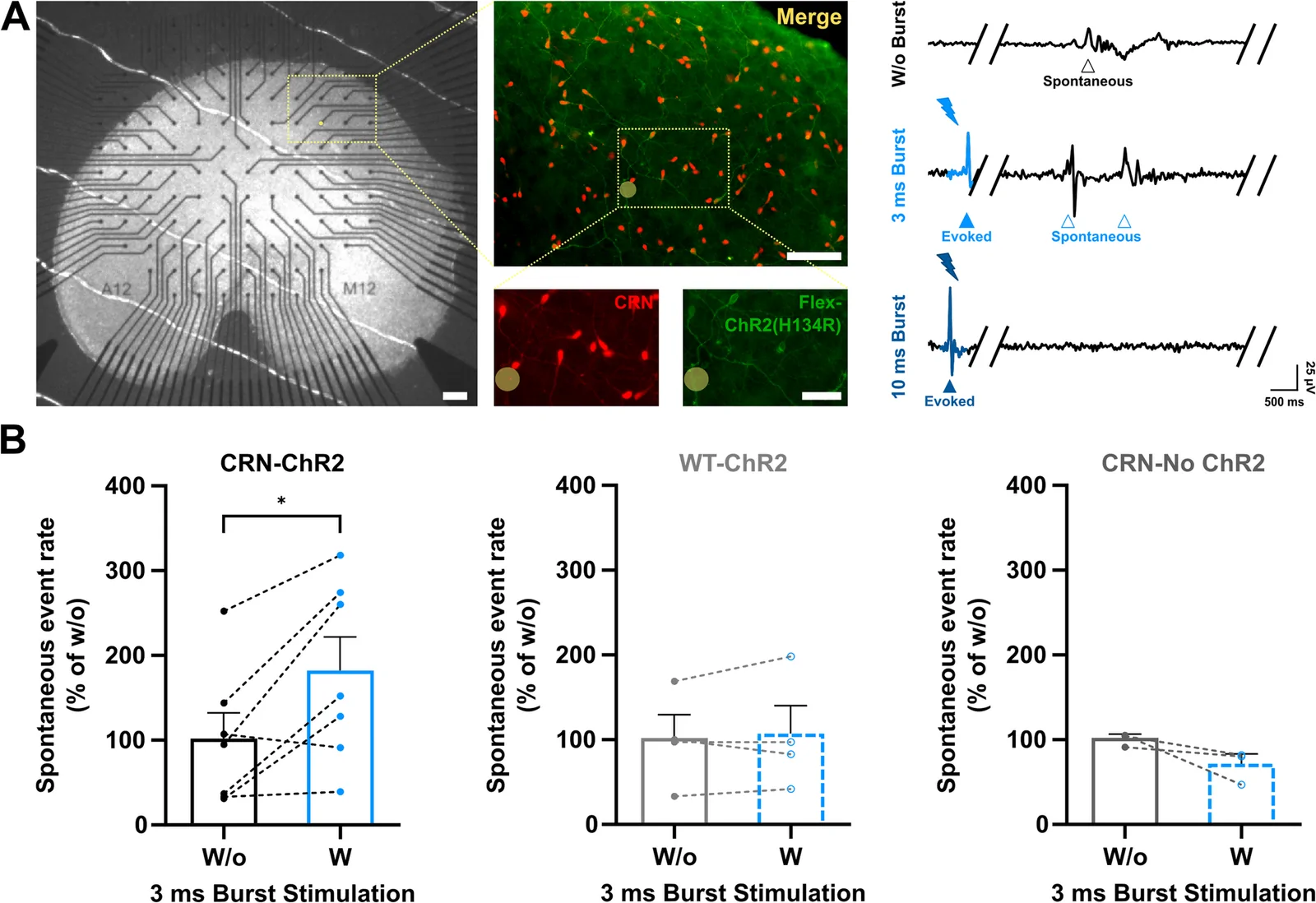
Electrophysiological analysis of cortical network activity during acute optogenetic stimulation of CRN.** A** Representative image of a cortical CRN-ChR2 OTC on MEA (left) with close-up showing tdTomato^+^ CRN (red), Flex-ChR2(H134R) AAV expression (green) and a selected MEA electrode (highlighted in ochre yellow). Scale bars: overview = 200 μm, insets = 50 μm. Representative field potential traces (right) from the highlighted electrode without and with optogenetic burst stimulation (3 and 10 ms pulses). Blue trace segments correspond to the periods during which burst stimulation was delivered. Filled triangles indicate stimulus-evoked responses, whereas empty triangles indicate the detected spontaneous events in-between repeated burst stimulations. Notably, 3 and 10 ms burst stimuli induced evoked responses. **B** Quantification of the spontaneous event rate (normalized to baseline event rate before stimulation) in-between repeated 3 ms burst stimulations for experimental (CRN-ChR2) and control conditions (WT-ChR2 and CRN-No ChR2). Repeated optogenetic activation of CRN-ChR2 significantly increased the frequency of spontaneous network events between optogenetic stimuli compared to control groups. *n* = 7 (CRN-ChR2), 4 (WT-ChR2), 3 (CRN-No ChR2) OTCs. *p < 0.05 (paired Student’s t-test)

The previous results indicate that CRN are deeply embedded into immature cortical networks via excitatory GABAergic inputs before their developmental loss during the second postnatal week. To address the functional implications of this CRN connectivity, the effects of targeted optogenetic CRN activation on cortical network activity and CRN survival rates were studied next. For this purpose, OTCs of ΔNp73Cre^+^/tdTomato^+^ mice were transduced with a Cre-dependent recombinant virus carrying the channelrhodopsin-2 H134R mutant variant-EYFP fusion protein (Flex-ChR2(H134R)-EYFP) to enable acute and chronic optogenetic activation of early and late CRN (Supplementary Fig. 4A). This resulted in a highly specific expression in ΔNp73-positive CRN (89 ± 2% of the total number of EYFP^+^ cells) while transduction efficiency amounted to 44 ± 4% of all ΔNp73-positive CRN at DIV7/8 (Supplementary Fig. 4A, B). An early, acute optogenetic light stimulation of CRN was performed at DIV7/8 under conditions that facilitate neuronal activity (0.5 mM Mg^2+^) (Supplementary Fig. 4C). Acute optogenetic stimulation with 20 Hz bursts comprising 3 or 10 ms light pulses was combined with electrophysiological MEA recordings to assess the direct and indirect effects of CRN activation on cortical network dynamics (Fig. 5A). Optogenetic stimulation of ΔNp73-positive CRN with a light pulse duration of 3 ms, resulted in rare light-evoked field responses in 4 out of 7 OTCs. In the responsive cultures only 9 ± 8% of all burst stimulations evoked immediate stimulus-evoked responses. By increasing the light pulse duration to 10 ms, light-evoked field responses were detectable in 4 out of 4 OTCs. In addition, the rate of direct responses upon burst simulation increased to 22 ± 11%.

Next, we analyzed the sustained effects of repeated CRN stimulation delivered every 20 s using 3 ms burst stimuli. Quantifying the frequency of spontaneous network-wide field oscillations during the intervals between successive 3 ms burst stimulations allowed us to assess the indirect effects of repeated CRN stimulation on cortical network excitability, independent of the rare, immediate, stimulus-dependent responses during stimulation (Fig. 5B). The relative event rate outside of stimulus time windows of ΔNp73-, ChR2-positive (CRN-ChR2) OTCs exposed to repeated CRN optogenetic activation was significantly (paired Student’s t-test, *p* = 0.02) increased (180 ± 40%) compared to baseline activity without light stimulation. In line with a sustained increase in excitability, spontaneous network events occurring in-between repeated burst stimulations had an amplitude of 104.7 ± 20.8 µV and involved in average 16.4 ± 2.9 electrodes. Both parameters were significantly (Wilcoxon test, *p* = 0.047 and paired Student’s t-test, *p* = 0.03, respectively) increased compared with unstimulated control conditions (83.5 ± 17.4 µV and 9.9 ± 2.6 electrodes). The increase in the number of participating electrodes per events was also reflected by a higher proportion of global spreading events (Supplementary Fig. 5).

This effect was absent in ΔNp73Cre^−^ wildtype cultures transduced with Flex-ChR2(H134) (WT-ChR2) and non-transduced ΔNp73Cre^+^ cultures (CRN-No ChR2) under repetitive burst stimulation (Fig. 5B). Thus, acute optogenetic stimulation of CRN in vitro increased the excitability of the cortical network, as reflected by evoked network responses during burst stimulations and increased frequency of spontaneous network-wide events between stimuli.

**Fig. 6:**
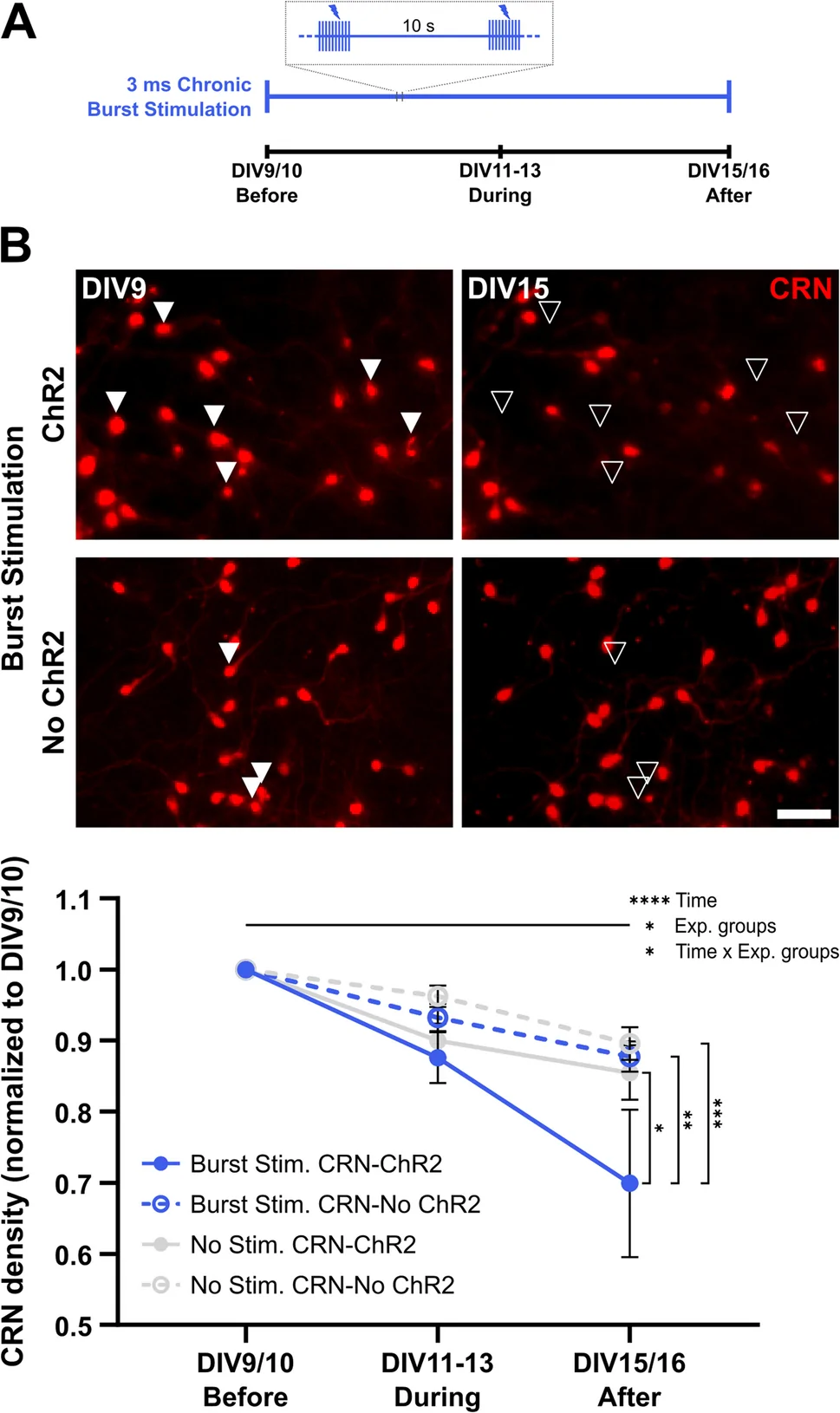
Longitudinal quantification of CRN survival during chronic optogenetic CRN stimulation in vitro.** A** Schematic timeline of the chronic optogenetic stimulus protocol. Burst stimulation with 10 light pulses lasting 3 ms at a frequency of 20 Hz was applied every 10 s from DIV9/10 to DIV15/16. Epifluorescence images were acquired before (DIV9/10), during (between DIV11 and DIV13) and at the end (DIV15/16) of the chronic stimulation to monitor CRN density. **B** Representative images of tdTomato^+^ CRN (red) in OTCs with and without ChR2 expression before and after the chronic optogenetic protocol (top). Filled triangles mark CRN that were present at DIV9 and subsequently eliminated before DIV15 (empty triangles). Quantification of CRN density (normalized to DIV9/10) at DIV11/13 and at DIV15/16 (bottom). Chronic stimulation of CRN for six days resulted in a significantly higher death rate of ∆Np73Cre^+^/tdTomato^+^ CRN compared to controls. Scale bar = 100 μm. *n* = 10 (Burst Stim. CRN-ChR2), 12 (Burst Stim. CRN-No ChR2), 8 (No Stim. CRN-ChR2), 14 (No Stim. CRN-No ChR2) OTCs. *p < 0.05 **p < 0.01, ***p < 0.001, ****p < 0.0001 (2-way RM ANOVA followed by Tukey’s post-hoc)

Next, we investigated the consequence of repeated CRN activation at later developmental stages, when CRN start to undergo developmental loss and lose their synaptic inputs. To this end, OTCs were subjected to chronic optogenetic stimulation for 6 days (144 h) between the second and third week in vitro (DIV9/10-DIV15/16) (Supplementary Fig. 4C). Here, optogenetic stimulation consisted of repeated light trains (10 stimuli with 3 ms-pulse duration and stimulus frequency of 20 Hz) every 10 s (Fig. 6A). Before (DIV9 or 10), during (between DIV11 and DIV13) and at the end (DIV15 or 16) of the chronic stimulation CRN density was assessed by repeated optical imaging in experimental (CRN-ChR2 OTCs with burst stimulation) and control groups (CRN-No ChR2 OTCs with burst stimulation, CRN-ChR2 OTCs without burst stimulation and CRN-No ChR2 OTCs without burst stimulation). The results showed that, although CRN density significantly decreased with time in every experimental group (2-Way RM ANOVA, Time *p* < 0.0001), resultant CRN densities over the course of the experiment significantly differed across experimental groups (2-Way RM ANOVA, Experimental groups *p* = 0.04). After 6 days of repeated optogenetic burst stimulation, normalized CRN density was significantly (2-Way RM ANOVA, Interaction *p* = 0.02) lower in the experimental group compared to control groups (Burst Stim. CRN-ChR2 = 0.7 ± 0.1, Burst Stim. CRN-No ChR2 = 0.9 ± 0.02, No Stim. CRN-ChR2 = 0.9 ± 0.04, No Stim. CRN-No ChR2 = 0.9 ± 0.02; Fig. 6B).

In summary, these results suggest that during early development CRN activation can shape cortical network dynamics and excitability. At later developmental stages, chronic CRN activation accelerates CRN cell loss.

## Discussion

Although CRN are well established as coordinators of cortical layering through reelin secretion, their functional integration into immature cortical networks largely remained unresolved. In the present study, we demonstrate that CRN receive dense GABAergic inputs from birth onwards, which, due to the persistent predominance of NKCC1 over KCC2, can activate CRN. A significant proportion of CRN are spontaneously active during cortical network events, and their activity correlates with that of both CRN and non-CRN neurons. Furthermore, with their predominant GABAergic input connectivity, CRN remain active when cortical network activity increases. During early development, CRN activation shapes cortical excitability and network dynamics, while at later developmental stages, chronic CRN activation accelerates CRN cell death. Thus, GABAergic excitation plays a dual role in CRN, supporting their functional integration into immature networks during early phases and promoting their developmental loss later on.

Both glutamatergic and GABAergic transmission undergo significant changes in synapse number, receptor subtype expression and postsynaptic response kinetics during the first postnatal weeks [54, 55]. However, while glutamatergic responses mediate a depolarizing membrane response throughout all developmental stages, a complete reversal in postsynaptic currents has only been observed for the GABAergic system during postnatal development [56, 57]. These functional changes in GABAergic transmission are paralleled by changes in the layer, cell type and even subcellular localization of GABAergic connections [43, 58]. The longitudinal profiling of GABAergic inputs to CRN in this study is based on three-dimensional spatial proximity between the presynaptic marker vGAT and the tdTomato reporter signal of CRN dendrites following an approach described previously [36]. Compared with other pre- and postsynaptic markers, vGAT can be detected from very early developmental stages, making it a valuable readout for longitudinal analyses [59–61]. Notably, during the early stages of development, when GABAergic synapse density in the cortex is low and mostly restricted to the upper layers [62], CRN dendrites are densely innervated by GABAergic synaptic inputs. In contrast to the progressive increase in GABAergic synapses across cortical layers during the first postnatal week (as shown here and in previous studies [42, 62]), the density of dendritic vGAT onto CRN remains consistently high while they are abundant, and even decreases considerably in mature, adult CRN. This is in line with previous functional and morphological studies showing prominent GABAergic synaptic inputs to CRN at early developmental phases [19, 21, 23, 28, 30]. The reduction of GABAergic synapses along CRN dendrites at later postnatal stages could be attributed to the described developmental transition of layer I (LI) GABAergic inputs from targeting disappearing CRN to targeting LII/III pyramidal neurons [20]. Indeed, the reduction of vGAT dendritic density takes place in the second postnatal week, when LI inhibitory circuits and higher-order sensory processing in the somatosensory cortex are established [46, 63, 64]. Accordingly, preventing CRN developmental loss alters this circuitry by decreasing sensory-driven inhibition of LII/III pyramidal cells [16, 20].

The early dense GABAergic innervation of CRN raises questions about its functional significance. In the immature cortex, neurons are integrated into dynamically changing, partially transient networks, in which the dominant spontaneous synchronized activity plays a key role for shaping neuronal migration, circuit assembly and connectivity, as well as synapse formation and maturation [44, 45, 65, 66]. Layer I is believed to constitute a temporary interface that receives afferent inputs and in turn sends outputs to target neurons, thereby underpinning columnar activity [23, 28, 67, 68]. Neocortical CRN have been previously reported to receive long-range synaptic inputs from layer V pyramidal neurons and layer VI reelin-positive interneurons, as well as local synaptic inputs within LI from inhibitory interneurons [19, 20, 30]. Our data show that during the first postnatal week, when the cortical network is still immature and undergoing developmental refinement, a meaningful proportion of CRN is spontaneously active. This is supported by calcium transients in OTCs at DIV7/8 and c-Fos immunoreactivity in slices ex vivo at P7/8. Importantly, although OTCs may represent a limited cortical model, and although we increased the occurrence of network events artificially by using low-Mg²⁺ conditions, the main characteristics of early network activity are preserved in these in vitro experiments [47, 69]. Furthermore, the functional integration of the CRN into this network activity aligns with c-Fos positivity under control conditions in acute slices. Future studies will need to investigate the extent to which CRN activity feeds back onto cortical network excitability in the immature cortex in vivo, and uncover the precise routes involved. The recordings in OTCs suggest that CRN calcium dynamics are highly temporally correlated with spontaneous cortical events at both single-cell and network level. Accordingly, prior works showed that developing LI contains overlapping networks of co-active cells in which CRN participate, and that this activity depends on synaptic transmission [27, 70]. Taken together, these findings support the idea that CRN participate in spontaneous network activity in the developing cortex and contribute to signal integration within LI. Furthermore, this stimulates the idea that CRN could serve as amplifiers of early network activity by providing feedback to lower-layer cortical networks. Thus, CRN network integration may be crucial during a developmental phase when neuronal migration, dendritic growth and synaptic wiring are significantly influenced by activity. Consequently, CRN are unlikely merely transient placeholders for GABAergic synapses, but could serve as active elements of the immature cortical network [44]. CRN activity has been reported to influence their own density in the neocortex through a secondary reallocation, which is essential for the proper assembly of LI architecture [71]. Moreover, the functional wiring of LI relies on crosstalk between spontaneous thalamic activity and CRN, with long-term consequences on cortical circuit organization [72]. As in other immature neurons [73, 74], GABA exerts an excitatory action on CRN [51]. The main mechanism underlying these depolarizing responses is the relative expression of the two chloride transporters NKCC1 and KCC2, that consequently influences the intracellular chloride concentration [75, 76]. In contrast to LII/III neurons, which undergo a developmental transition towards hyperpolarizing GABA signaling during the first postnatal week (see this study and [77]), NKCC1 expression remains higher than KCC2 in CRN at early stages [51, 52]. This particular expression profile in CRN is retained beyond developmental periods, even in persistent CRN at late adulthood. This suggests that the depolarizing action of GABA is not a transient feature of early CRN, but rather a stable property of this cell type. Moreover, the limited functional maturation of glutamatergic inputs onto CRN [19, 23, 26, 30] may contribute to their persistent low KCC2 expression. Given that KCC2 regulation is activity- and calcium-dependent [78–81], reduced depolarizing drive could impair its developmental upregulation. Future studies directly assessing or manipulating excitatory synaptic activity in CRN will be required to test this hypothesis. Functionally, the persistent GABA-mediated depolarization can keep CRN responsive to local GABAergic input, allowing these neurons to be recruited by ongoing network activity rather than suppressed by it. Our observation that an acute, massive increase in network activity induced by 4-AP application was associated with a slight increase in c-Fos immunoreactivity in CRN further supports this suggestion. In this way, depolarizing and putatively excitatory GABAergic inputs represent the major mechanism through which CRN can be activated and potentially influence early network dynamics.

CRN are glutamatergic neurons [4, 82, 83], but our understanding of their output connectivity is limited. Labeling studies have suggested that cortical CRN innervate LII/III and LV pyramidal neurons, and may also contact other CRN in LI not only via chemical synapses but also via gap junctions, although findings regarding the latter are not fully consistent [23, 30]. Nevertheless, the postsynaptic targets of CRN and their influence on cortical network are still largely unknown and constitute an active line of research. In order to assess the effects of CRN activation on early networks we recurred to electrophysiological recordings of cortical OTCs in parallel with acute optogenetic stimulation of transgenic CRN. In the hippocampus, where CRN input-output connectivity has been better described [84, 85], a CRN-specific optogenetic approach revealed electrophysiological responses in both GABAergic interneurons and pyramidal cells, highlighting a hippocampal feedforward circuit driven by CRN [82]. In the neocortex, optogenetic photostimulation of rescued CRN has previously been attempted by crossing BaxCKOΔNp73Cre mice with Cre-dependent ChR2-expressing mice. However, no detectable responses were observed [16]. In the present study, optogenetic stimulation of CRN in OTCs is enabled by an AAV-based Cre-specific overexpression approach with the channelrhodopsin-2 (ChR2) H134R variant. For photostimulation, a repeated burst protocol consisting of 10 light pulses delivered at 20 Hz was applied to mimic the typical burst pattern and allow reliable action potential induction in immature cortical neurons [40, 86, 87] while minimizing the risks of opsin desensitization, depolarization block, and synaptic depression [88, 89]. Repeated optogenetic activation of CRN resulted in an overall increase in the excitability of the OTCs network, with a higher frequency, amplitude and spread of spontaneous network events occurring outside stimulus windows. Additionally, we recorded stimulus-evoked field responses, the occurrence and reliability of which increased with longer pulse duration (10 ms vs. 3 ms). These findings indicate that the functional integration of CRN into immature cortical circuits could serve to modulate network activity on a broad level. While the extent of electrical coupling between CRN remains to be determined, this necessarily would affect the spatial extent of CRN-mediated effects on cortical network activity [90]. In this context, CRN may contribute to the amplification and coordination of early spontaneous activity, and thereby participate in the establishment of immature cortical dynamics and cortical development beyond their established role as a source of reelin. Previous studies hypothesized that CRN projections onto the terminal tufts of LII/III and LV pyramidal neurons may modulate the Ca^2+^ spikes that integrate synaptic activity of developing pyramidal cells contributing to the establishment of cortical domains [30]. Furthermore, the activity of CRN may be synchronized by GABAergic interneurons, presumably Martinotti cells [91]. Although not addressed in the present study, it will be interesting to investigate whether CRN possess intrinsic pacemaker properties that drives neocortical activity at early stages.

The fact that CRN can act as active participants in early immature circuits, together with the established role of neuronal activity in cortical development and in the elimination of specific CRN subtypes, raises an important question about their role in their own transient nature, and therefore in final architecture of cortical networks. Indeed, although neuronal activity generally excerpts an anti-apoptotic effect on developing cortical neurons [92, 93], (septum-derived) CRN actually die in an activity-dependent manner [9, 16, 35]. Supporting these findings, late chronic optogenetic activation of ΔNp73-positive CRN accelerates their death, suggesting that CRN activity actively contributes to their transience in the neocortex. The putative depolarizing action of GABAergic inputs to CRN likely contribute to their activity-induced disappearance, as pharmacological block of NKCC1 increased survival rates of CRN (this study and [35]). Beyond its synaptic effects, NKCC1-mediated ion influx has been implicated in osmotic imbalance, cellular swelling, and neuronal injury in other cell types under severe pathological conditions [94, 95]. However, the relevance of these effects in CRN remains to be determined. The precise timing of CRN demise has important clinical implications. Hence, aberrant survival of CRN leads to an E/I imbalance due to increased excitatory drive [15, 16, 20]. While this necessarily affects early cortical activity, increased CRN density has also been documented in human cortical pathologies, such as Ammon’s horn sclerosis, focal cortical dysplasia, and polymicrogyria in adults [96–98]. Conversely, premature loss of CRN results in long-lasting morphological defects in LI architecture and a reduction in the E/I ratio [71], which has been associated with neuropsychiatric pathologies such as autism spectrum disorder (ASD), Rett syndrome and fragile X syndrome [99].

In conclusion, this study reveals characteristic features of CRN throughout their lifespan, shedding light on new facets of their functional integration into immature neocortical networks. The putative excitatory GABAergic drive and the functional integration of CRN into immature networks ultimately appear to play a role in their transient nature. However, this study also shows that CRN activity not only correlates with early cortical network activity but can interfere with it, pointing towards a self-amplifying CRN-network loop that increases cortical activity levels during early development and leads to the natural loss of CRN later on.

## Supplementary Information

Supplementary Material 1.

## Data Availability

No datasets were generated or analysed during the current study.
