# Supplementary material for: Network integration of neocortical Cajal-Retzius neurons shapes early cortical dynamics and contributes to their transient nature
Source: Cell Commun Signal. 2026 Sep 9;24:482. doi: 10.1186/s12964-026-03210-6 (PMC13556013; doi:10.1186/s12964-026-03210-6)
Supplement: Supplementary file 1 [file 12964_2026_3210_MOESM1_ESM.docx]

**Network Integration of Neocortical Cajal-Retzius Neurons Shapes Early Cortical Dynamics and Contributes to Their Transient Nature** - *Federico De Rosa, Werner Kilb, Heiko J. Luhmann, Anne Sinning^*^*

**Supplementary Figures**

**Supplementary Figure 1 | Distribution of putative GABAergic synapses onto CRN and in the upper neocortical layers throughout development. (A)** Representative reconstructions of single CRN (red) at P3 and P17 with identified corresponding dendritic vGAT-positive spots (gray) and relative close-ups. Scale bars: overview = 10 µm, insets = 2 µm. **(B)** Representative images of vGAT immunostaining (gray) and tdTomato reporter signal of CRN (red) at P3 and P17 and quantification of vGAT-positive spots density in the upper layers of the cortex from P3 to adulthood. Consistent with the maturation of the GABAergic synaptic connectivity, vGAT density increased from P3 to P17 and remained stable into adulthood. n = 6 (P3), 7 (P8), 8 (P17), 5 (BaxCKO>12w) FOV from at least 4 slices per group. Scale bar = 20 µm. **p* < 0.05 (Kruskal-Wallis test).

**Supplementary Figure 2 | Quantification of NKCC1 and KCC2 in CRN and layer II/III neurons across development.** NKCC1 and KCC2 single-plane puncta per cell in CRN and layer II/III neurons at P3, P8, P17, and in adulthood (>12 weeks using the BaxCKO∆Np73Cre^+^ lineage). NKCC1 and KCC2 levels remained stable in CRN across development, while both transporters increased their expression in layer II/III neurons, particularly KCC2. n = 32 (P3), 31 (P8), 27 (P17), 32 (BaxCKO>12w) CRN; n = 32 (P3), 31 (P8), 29 (P17), 33 (BaxCKO>12w) LII/III neurons from 3 mice per time point. ***p* < 0.01, *****p* < 0.0001 (Kruskal-Wallis test).

**Supplementary Figure 3 | Monitoring of survival of ∆Np73^+^ CRN** **after bumetanide treatment in vitro.** Representative images of tdTomato^+^ CRN (red) from OTCs at DIV4 and DIV12, either untreated controls or chronically treated with bumetanide (10 µM) from DIV1 onward, with longitudinal quantification of CRN density (normalized to DIV4-6) from DIV4 to DIV12 (shown below). Note the trend towards increased CRN densities at later developmental stages following bumetanide treatment, compared with the progressive decline in CRN density in untreated control OTCs. Scale bar = 100 µm. n = 15 (control), 10 (bumetanide-treaded) OTCs. *****p* < 0.0001 (2-way RM ANOVA).

**Supplementary Figure 4 | Validation of Cre-dependent channelrhodopsin-2 expression in ∆Np73Cre^+^ CRN and acute and chronic optogenetic protocols. (A)** Representative images of tdTomato^+^ CRN (red) and GFP immunostaining of Flex-ChR2(H134R)-EYFP AAV (green) in ∆Np73Cre^+^ OTCs at DIV7, DIV9 and DIV15. Scale bar = 20 µm. **(B)** Assessment of Flex-ChR2(H134R) AAV transduction at DIV7/8. Results revealed high specificity (transduced CRN / total number of transduced cells) for CRN (~ 90%) and good efficiency (~ 45%; calculated as transduced CRN / total tdTomato^+^ CRN) upon AAV-based transduction at DIV0. **(C)** Experimental timeline of acute and chronic stimulation of CRN. Transduced OTCs were assigned to either an acute or a chronic stimulation paradigm. Combined MEA recordings and acute optogenetic burst stimulation of CRN (10 pulses of 3/10 ms at a frequency of 20 Hz frequency, every 20 s) were performed at DIV7/8. For the chronic protocol, OTCs were exposed to repeated optogenetic burst stimulation (10 pulses of 3 ms at a frequency of 20 Hz, every 10 s) from DIV9/10 to DIV15/16 and CRN density was tracked through epifluorescence imaging. n = 7 (DIV7/8) OTCs.

**Supplementary Figure 5 | Characterization of the effects of repeated optogenetic stimulation of CRN on spontaneous cortical network events. (A, B)** Quantification of the amplitude and number of electrodes involved in spontaneous events occurring between repeated 3 ms burst stimulations. Repeated optogenetic activation of CRN-ChR2 significantly increased both parameters compared with the unstimulated control condition. **(C, D)** Quantification of the relative occurrence of local and global events and the relative number of electrodes involved in these events. The relative proportion of local non-spreading events (< 5 active electrodes) did not change significantly with repeated optogenetic activation of CRN-ChR2, whereas the relative proportion of global spreading events (> 10 active electrodes) significantly increased. The relative number of electrodes involved in local and global events (number of participating electrodes / total number of active electrodes per recording) remained unchanged. n = 7 CRN-ChR2 OTCs. **p*<0.05 (paired Student’s t-test or Wilcoxon test).
